# Study on clinical application of susceptibility weighted imaging ombined with diffusion weighted imaging in patients with Liver Cirrhosis complicated with small Hepatocellular Carcinoma

**DOI:** 10.12669/pjms.37.3.3822

**Published:** 2021

**Authors:** Zhi-bo Hou, Fei Zhao, Bin Zhang, Chun-zhu Zhang

**Affiliations:** 1 Zhi-bo Hou, Medical Imaging Center, Peking University Shougang Hospital, Beijing, 100144, China; 2 Fei Zhao, Department of Imaging, Aerospace Center Hospital, Beijing, 100049, China; 3 Bin Zhang, Medical Imaging Center, Peking University Shougang Hospital, Beijing, 100144, China; 4 Chun-zhu Zhang, Department of Radiology, Eye Hospital, China Academy of Chinese Medical Sciences, Beijing, 100040, China

**Keywords:** Susceptibility weighted imaging, Diffusion weighted imaging, Liver cirrhosis, Small hepatocellular carcinoma, Diagnosis

## Abstract

**Objectives:**

To evaluate the clinical value of susceptibility weighted imaging (SWI) combined with diffusion weighted imaging (DWI) in patients with liver cirrhosis complicated with small hepatocellular carcinoma (SHCC).

**Methods:**

A total of 40 patients with liver cirrhosis and 44 nodules were treated with conventional nuclear magnetic scanning (T1WI, T2WI) and SWI combined with DWI; the results were judged by two senior physicians; the t test, χ^2^ test, rank sum test, and other methods were used for contrastive analysis of the pathological results of different scanning methods after operation or puncture.

**Results:**

Contrast analysis of the different MRI scanning methods and pathological results showed that among the 32 nodules of small hepatocellular carcinoma, 24 cases were diagnosed by conventional MRI, with the coincidence rate being 75%, 30 cases were diagnosed by SWI DWI, with the coincidence rate being 96%; significant difference was found between the two groups (p=0. 04). Significant differences were found in the specificity, sensitivity and accuracy of different scanning methods in the diagnosis of small hepatocellular carcinoma (specificity, accuracy, p=0.04; sensitivity p=0.01). The SWI of small hepatocellular carcinoma nodules showed hyperintensity, and the degree of iron deposition was low. Significant difference was found between small hepatocellular carcinoma nodules and other nodules (comparison of SWI signal degree, p=0.01; comparison of iron deposition degree, p=0.00).

**Conclusion:**

The SWI of small hepatocellular carcinoma nodules showed hyperintensity, and the degree of iron deposition was low. The coincidence rate of SWI+DWI scanning is higher than that of conventional scanning methods in the diagnosis of small hepatocellular carcinoma, and the difference in specificity, sensitivity and accuracy has obvious advantages. SWI+DWI scanning can improve the detection rate of liver cirrhosis complicated with small hepatocellular carcinoma.

## INTRODUCTION

Liver cirrhosis is a common chronic disease in digestive system, which is mostly caused by hepatitis B virus infection.^1^ In the advanced stage, it gradually develops into hepatic encephalopathy, upper gastrointestinal hemorrhage, carcinogenesis and so on, which causes great harm to the patients. Primary hepatic carcinoma is one of the most serious complications in patients with liver cirrhosis.^2^ Early diagnosis is of great importance for the treatment effect and prognosis of patients. KWON’s study showed that.^3^ iron deposition occurred in non-cancerous nodules before carcinogenesis after cirrhosis of liver, and iron deposition in nodules after carcinogenesis gradually decreased.

SWI is a magnetic resonance scanning method, and it is of certain significance to detect iron deposition in tissues.^4^ DWI also has higher diagnostic effect in the diagnosis and grading of liver cancer.^5^ We use SWI combined with DWI to analyze the carcinogenesis of nodules under the background of liver cirrhosis, suggesting that the combination of the two is significantly better.

## METHODS

### Ethical Approval:

The study was approved by the Institutional Ethics Committee of Peking University Shougang Hospital (Septemebr 17^th^ 2020), and written informed consent was obtained from all participants.

### Inclusion criteria:

1. Patients who meet the diagnostic criteria for liver cirrhosis^6^;
2. Patients who have received conventional MRI imaging and other imaging methods such as DWI and SWI.
3. Patients with single nodule diameter < 3cm or the number of nodules less than 2, the sum of diameters < 3 cm, meeting the diagnostic criteria for small hepatocellular carcinoma^7^;
4. Patients with definite pathological diagnosis results (the source of specimens includes surgical specimens and biopsy specimens).

### Exclusion criteria:

1. Patients with incomplete clinical or pathological data;
2. Patients with no liver cirrhosis confirmed by pathology;
3. Patients with mental illness or other cognitive impairment who could not cooperate with the completion of the study. All patients agreed to participate in the study and signed the informed consent.

The clinical data of 40 patients with liver cirrhosis admitted to the hospital were analyzed retrospectively, including 22 males and 18 females, aged 42~76 years old, with an average age of 57.73±10.42 years old. A total of 44 nodules for the 40 patients were examined by pathologist; the diameter of the lesions was 0.7-2.8cm, with an average of (2.1±0.7) cm. A total of 31 lesions for 30 patients were post-operation specimens, and a total of 13 lesions for 10 patients were biopsy specimens. There were 32 nodules of liver cirrhosis complicated with small hepatocellular carcinoma and 12 other nodules diagnosed pathologically.

### MRI scanning method:

MRI was performed by SIEMENS 3.0T magnetic resonance imaging system. The scanning items included T1WI, T2WI and other conventional plain scan enhancement, SWI, and DWI. Patients were prohibited from drinking water in four hours before examination, and gadopentetate dimeglumine was used as enhancer. MR syringe was used, the dose was 30ml and it was rated at 2ml/s; the arterial phase, portal venous phase and equilibrium phase were delayed for 20-25s, 60-65s and 180-200s respectively. 2D sequence was used for SWI; parameters: slice thickness 5mm, slice spacing 1mn, time of repetition (TR) 150ms, time of echo (TE) 10ms, reverse angle 20 degrees, field of view (FOV) 285mm×350mm~330mm X350mm, matrix 187× 384-168 ×320. After scanning, the image was reconstructed by using software.

### Image interpretation:

Two senior physicians in our department read the images by single-blind technique (the physicians did not know the patient data and information). The contents of the image reading include the following:

(1) Whether there is iron deposition in the liver^8^ (iron deposition in the liver is characterized by focal speckle hypointensity scattered in the liver relative to the background liver tissue or decrease in the liver signal intensity relative to the paravertebral muscles), as well as the degree of deposition; criteria: Semi-quantitative analysis of iron deposition is performed with reference to the criteria proposed by Cotes, et al.^9^: Grade 0 means no iron deposition; Grade-I means a small amount of iron deposition, and sparse light blue iron staining positive area can be seen in the liver; Grade-II is mild iron deposition, iron staining area is scattered and focal; Grade-III is moderate iron deposition, iron staining positive area is scattered and it is in multiple distribution state; grade 4 is severe iron deposition, iron staining positive area is in dispersive distribution state; (2) Whether there is canceration in intrahepatic nodules under conventional sequence (T1WI, T2WI and other conventional plain scan enhancement);

(3) Whether there is canceration in intrahepatic nodules under SWI + DWI sequence^10^ (in the background of iron deposition in liver, nodular hyperintense iron devoid area is determined as HCC; there is iron deposition in the nodules, but nodular iron devoid areas (nodule in nodule) are observed, it is also judged to be HCC; without liver iron deposition background, it is determined as uncertain nodules by SWI, and the diagnosis is still based on conventional MRI).

### Observation indicators:

(1) Contrastive analysis of pathological results and MRI routine sequence and SWI+ DWI sequence; (2) Comparison of the sensitivity and specificity in the diagnosis of cirrhotic nodules under conventional MRI sequence and SWI+ DW sequence; (3) The relationship between nodular properties and 3SWI signal, iron deposition degree.

### Statistical method:

All the data were processed by using the SPSS 20.0, and the measurement data were expressed by (χ̄ ±S). The data between the groups were analyzed by t test for two groups of independent samples, and χ^2^ test was used for fate comparison; rank sum test was used for ranked data; P<0.05 means the difference is statistically significant.

## RESULTS

Contrastive analysis of the different MRI scanning methods and pathological results are shown in TableI. Among the 32 nodules of small hepatocellular carcinoma, 24 cases were diagnosed by conventional MRI, the coincidence rate was 75%, 30 cases were diagnosed by SWI DWI, the coincidence rate was 96%; significant difference was found between the two groups (p=0. 04); this suggests that the coincidence rate of SWI+DWI scanning is higher than that of conventional scanning.

**Table I:** Contrastive analysis of the different MRI scanning methods and pathological results coincidence rate (χ̄ ±S) n=32.

Group	Diagnosed	Undiagnosed	Coincidence rate *
Conventional MRI group	24	8	75%
MRI group			
SWI+ DWI group	30	2	96%
χ^2^			4.26
P			0.04

* P < 0.05

Significant differences were found in the specificity, sensitivity and accuracy of different scanning methods in the diagnosis of small hepatocellular carcinoma (specificity, accuracy, p=0.04; sensitivity p=0.01); SWI+DWI had obvious advantages in the diagnosis of small hepatocellular carcinoma (Table-II)

**Table II:** Contrastive analysis of diagnostic specificity, sensitivity and accuracy of different MRI scanning methods (χ̄ ±S) n=44.

Group	Specificity (%) *	Sensitivity (%) *	Accuracy (%) *
Conventional MRI,	81.24	83.71	80.34
SWI+ DWI	91.26	93.55	91.47
χ^2^	4.35	4.79	4.43
p	0.04	0.01	0.04

* P<0.05.

The relationship between the signal intensity of SWI and the degree of iron deposition and the properties of nodules is shown in Table-III; significant difference was found between small hepatocellular carcinoma nodules and other nodules (SWI, p=0.01; comparison of iron deposition degree, p=0.00); The SWI of small hepatocellular carcinoma nodules showed hyperintensity, and the degree of iron deposition was low.

**Table III:** Contrastive analysis of nodular properties and 3SWI signal, iron deposition degree (χ̄ ±S) n=44.

	SWI signal intensity (case %) *				Degree of iron deposition (case %) *		

Group	Number of cases	Low	Middle	High	Grade-I	Grade-II	Grade-III
Small hepatocellular carcinoma nodules	32	2 (6.25)	5 (15.63)	25 (78.12)	27 (84.38)	2 (6.25)	3 (9.37)
Nodules of other nature	12	5 (41.67)	3 (25)	4 (33.33)	2 (16.67)	6 (50)	4 (33.33)
Z		13.13				14.47	
p		0.01				0.00	

* P<0.05

## DISCUSSION

Hepatocellular carcinoma (HCC) is very common in clinic and its mortality is high. Studies found^11^ that 80%~90% of HCC patients had a background of liver cirrhosis, and HCV infection is the most common cause.^12^ The prognosis of advanced HCC is poor and the survival rate is low. Ultrasound and alpha-fetoprotein examination are the most important auxiliary diagnosis[13]. However, the survival rate of the patients was higher when the diagnosis and treatment of the disease were performed in the early stage.^14^ Therefore, it is very important to identify the carcinogenesis of small nodules in the background of liver cirrhosis.^15^

MRI is a common diagnostic method for liver cancer. SWI is a magnetic contrast enhancement technique, which can be used to detect the magnetic susceptibility of different tissues. In this study, SWI technique was used to detect the degree of iron deposition in the patients with liver cirrhosis nodules, so as to explore the role of SWI in the differentiation of the nature of cirrhotic nodules, and to provide clinical basis for the diagnosis of carcinogenesis of cirrhotic nodules. Hsu, et al. found that^16^ SWI had the potential to distinguish the grade of hepatocellular carcinoma. Yang’s study further confirmed that^17^ there was a significant correlation between the SWI sensitivity signal intensity and the density of histologic microvessels (r = 0.753, P < 0.001).

The study of Li RK and its colleagues^18^ confirmed that 89 hepatocellular nodules in 68 patients with liver cirrhosis showed SWI sensitivity, specificity, and accuracy; positive predictive value (PPV) and negative predictive value (NPV) were 84.4% and 84.4%, 91.7% and 75%, 85.4% respectively, suggesting that SWI could reflect the decrease of iron content in liver cancer and had a higher advantage in the diagnosis of malignant nodules of liver cirrhosis. Ruo et al.^19^ found that SWI could accurately provide valuable information about hepatocellular carcinoma, compared with T1WI, T2WI and T2. Our study showed that there was a correlation between the nodular properties and SWI signal intensity, and iron deposition degree; significant difference was found between small hepatocellular carcinoma nodules and other nodules (SWI, p=0.01; comparison of iron deposition degree, p=0.00); the SWI of small hepatocellular carcinoma nodules showed hyperintensity, and the degree of iron deposition was low. This is similar to that reported in the literatures.

DWI is also of great significance in the diagnosis of liver cancer. Kim’s study has confirmed that DWI was helpful in distinguishing atypical nodules from small hepatocellular carcinoma in patients with liver cirrhosis.^20^ DWI can also be used to distinguish cholangiocarcinoma, liver capsule tumor and determine the nature of the interval in cysts.^21^ High contrast DWI is more valuable in the diagnosis of liver cancer by image registration.^22^ Our study showed that among the 32 nodules of small hepatocellular carcinoma, 24 cases were diagnosed by conventional MRI, the coincidence rate was 75%, 30 cases were diagnosed by SWI DWI, the coincidence rate was 96%; significant difference was found between the two groups (p=0. 04); this suggests that the coincidence rate of SWI+DWI scanning is higher than that of conventional scanning. Significant differences were found in the specificity, sensitivity and accuracy of SWI+DWI and conventional MR scanning in the diagnosis of small hepatocellular carcinoma (specificity, accuracy, p=0.04; sensitivity p=0.01), and it had obvious advantages in the diagnosis of small hepatocellular carcinoma.

### Limitations of this study:

1. The sample size is small; 2. Retrospective analysis is conducted for the patient data and no prospective study is carried out; 3. There is no study to distinguish the liver function and the degree of liver cirrhosis of the patients. Only cancerous nodules are compared with non-cancerous nodules. We are also further collecting cases and clinical data, and starting prospective analysis of some patients, to further improve the study.

## CONCLUSION

The coincidence rate of SWI+DWI scanning is higher than that of conventional scanning methods in the diagnosis of small hepatocellular carcinoma, and the difference in specificity, sensitivity and accuracy has obvious advantages. It can improve the detection rate of liver cirrhosis complicated with small hepatocellular carcinoma.

### Authors’ Contributions:

**ZBH & FZ:** designed this study and prepared this manuscript,and are responsible and accountable for the accuracy or integrity of the work.

**CZZ:** Collected and analyzed clinical data;

**BZ:** Significantly revised this manuscript.

**ZBH & FZ:** Both contributed this manuscript equally.
